# Stress-Induced Microspore Embryogenesis Requires Endogenous Auxin Synthesis and Polar Transport in Barley

**DOI:** 10.3389/fpls.2019.01200

**Published:** 2019-09-27

**Authors:** Yolanda Pérez-Pérez, Ahmed-Abdalla El-Tantawy, María Teresa Solís, María C. Risueño, Pilar S. Testillano

**Affiliations:** ^1^Pollen Biotechnology of Crop Plants Group, Biological Research Center, CIB-CSIC, Madrid, Spain; ^2^Department of Genetics, Physiology and Microbiology, University Complutense of Madrid, Madrid, Spain

**Keywords:** auxin, cell reprogramming, microspore embryogenesis, kynurenine, α-(*p*-chlorophenoxy)-isobutyric acid, N-1-naphthylphthalamic acid, barley, *Hordeum vulgare*

## Abstract

Stress-induced microspore embryogenesis is a model *in vitro* system of cell reprogramming, totipotency acquisition, and embryo development. After induction, responsive microspores abandon their developmental program to follow an embryogenic pathway, leading to *in vitro* embryo formation. This process is widely used to produce doubled-haploid lines, essential players to create new materials in modern breeding programs, particularly in cereals, although its efficiency is still low in many crop species, because the regulating mechanisms are still elusive. Stress signaling and endogenous hormones, mainly auxin, have been proposed as determinant factors of microspore embryogenesis induction in some eudicot species; however, much less information is available in monocot plants. In this study, we have analyzed the dynamics and possible role of endogenous auxin during stress-induced microspore embryogenesis in the monocot *Hordeum vulgare*, barley. The results showed auxin accumulation in early proembryo cells, from embryogenesis initiation and a further increase with embryo development and differentiation, correlating with the induction and expression pattern of the auxin biosynthesis gene *HvTAR2-like*. Pharmacological treatments with kynurenine, inhibitor of auxin biosynthesis, and α-(*p*-chlorophenoxy)-isobutyric acid (PCIB), auxin antagonist, impaired embryogenesis initiation and development, indicating that *de novo* auxin synthesis and its activity were required for the process. Efflux carrier gene *HvPIN1-like* was also induced with embryogenesis initiation and progression; auxin transport inhibition by N-1-naphthylphthalamic acid significantly reduced embryo development at early and advanced stages. The results indicate activation of auxin biosynthesis with microspore embryogenesis initiation and progression, in parallel with the activation of polar auxin transport, and reveal a central role of auxin in the process in a monocot species. The findings give new insights into the complex regulation of stress-induced microspore embryogenesis, particularly in monocot plants for which information is still scarce, and suggest that manipulation of endogenous auxin content could be a target to improve *in vitro* embryo production.

## Introduction

The ability of plant cells to regenerate organs and embryos through *in vitro* culture is a clear example of the high plasticity of the plant kingdom, a property that has been extensively applied in plant biotechnology for propagation, conservation, and breeding (Germana and Lambardi, 2016) of numerous species of interest in agriculture, forestry, and industry. *In vitro* embryogenesis has been induced in a wide range of cell types, including haploid microspores, which can acquire totipotency and embryogenic competence by appropriate inductor factors, giving rise to an entire embryo (Feher, 2015;Testillano et al., 2018a). During *in vivo* anther development, microspores develop and follow the gametophytic pathway to produce pollen grains. *In vitro*, isolated microspores, at the responsive stage of vacuolated microspore, can be reprogrammed by stress treatments, becoming totipotent cells and producing haploid and doubled-haploid (DH) embryos and plants. This process, known as stress-induced microspore embryogenesis, is widely used in plant breeding to rapidly obtain DH plants, which represent a source of new genetic variability, fixed in complete homozygous plants in only one generation step (Maluszynski et al., 2003; Kumlehn and Stein, 2014). Production of DH lines is currently a standard method of the creation of new material in many modern breeding programs, although its application is limited in many crop species due to low efficiency. Stress-induced microspore embryogenesis is also a convenient model system to analyze the molecular mechanisms underlying cell reprogramming, totipotency, and embryogenesis, particularly in systems where induction occurs through isolated microspore cultures.

Despite knowledge gained in recent years, the complex regulatory network of cell reprogramming leading to embryogenesis is still far to be fully elucidated. Stress signaling, hormones, and epigenetic mechanisms have been proposed as crucial factors underlying totipotency acquisition and embryogenesis induction in microspores (Testillano, 2019), as well as in somatic embryogenesis from other cell types (Feher, 2015; Díaz-Sala, 2018). The effects of plant hormones on *in vitro* embryogenesis initiation and progression are not well understood. Many somatic embryogenesis systems are induced by exogenous hormone treatments, mainly auxins. On the contrary, microspore embryogenesis is induced by stress, like temperature, starvation, or osmotic treatment (Touraev et al., 1996; Maluszynski et al., 2003), without addition of hormones in the culture media. The main model systems for stress-induced microspore embryogenesis are established in *Brassica napus* (dicot) and *Hordeum vulgare* (monocot), through isolated microspore cultures in media without exogenous auxins (Kasha and Kao, 1970; Kumlehn and Stein, 2014). Therefore, stress-induced microspore embryogenesis in these systems constitutes a very appropriate model to analyze endogenous hormone function during *in vitro* embryogenesis initiation and progression.

Auxin is the most significant hormone in plant growth, with a key role in regulation of cell division and differentiation (Weijers et al., 2018). Auxins, specifically its major form, indole–acetic acid (IAA), are involved in numerous developmental processes (Petrasek and Friml, 2009; Moreno-Risueño et al., 2010; Leyser, 2018; Wang and Jiao, 2018), including embryogenesis (Möller and Weijers, 2009), being auxin biosynthesis upregulated throughout zygotic embryo development. Major auxin biosynthesis, transport, and signaling pathways have been dissected in the last decades in the eudicot model species *Arabidopsis* (Mironova et al., 2017; Leyser, 2018). Although less information on auxin is available in monocots, studies in maize and rice have shown an important degree of conservation of auxin pathways between eudicot and monocot species (McSteen, 2010; Forestan and Varotto, 2012; Balzan et al., 2014). Several pathways have been described for auxin biosynthesis, being the indole-3-pyruvic acid (IPA) pathway the major route in most eudicot and monocot species (McSteen, 2010; Zhao, 2014). In this two-step route, the tryptophan aminotransferase of *Arabidopsis* 1 (TAA1) and tryptophan aminotransferases-related 1 and 2 (TAR1, TAR2) convert the amino acid tryptophan to IPA; subsequently, flavin monooxygenases of the YUCCA family (YUC) catalyze the conversion of IPA to IAA (Brumos et al., 2014; Zhao, 2014). TAA1/TAR and YUC genes play critical roles in many plant developmental processes and particularly in embryogenesis of both eudicot and monocot plants (Zhao, 2014; Shao et al., 2017). An efficient method to explore the role of TAA1/TAR-dependent auxin biosynthesis has been the use of -kynurenine (Kyn), a small molecule that competitively inhibits TAA1/TAR activity (He et al., 2011), with reported inhibitory effects of auxin biosynthesis in a range of auxin-related processes (de Wit et al., 2015; Nomura et al., 2015). It is well established that auxin action depends on its local biosynthesis and polar transport between cells, where efflux carrier proteins of the pinformed family (PINs) play a key role (Petrasek and Friml, 2009; Adamowski and Friml, 2015; Bennett, 2015). Among the canonical PINs, PIN1 has a central function during embryogenesis (Zazimalova et al., 2010; Prasad and Dhonukshe, 2013). Evidence of the important role of auxin transport in development has been obtained by the use of inhibitors of polar auxin transport (PAT), like N-1-naphthylphthalamic acid (NPA). Treatment with NPA has been reported to cause defects in vegetative and reproductive development, including embryogenesis, in eudicots and monocots (Wu and McSteen, 2007; Larsson et al., 2008; McSteen, 2010; Prasad and Dhonukshe, 2013). Other commonly used auxin inhibitor is α-(*p*-chlorophenoxy)-isobutyric acid (PCIB), which inhibits auxin action and its physiological effects (Xie et al., 2000).

Regarding *in vitro* embryogenesis systems, several studies have shown endogenous auxin accumulation during early microspore embryogenesis in a few dicot species, *B. napus* and *Quercus suber* (Prem et al., 2012; Rodríguez-Sanz et al., 2014; Rodríguez-Sanz et al., 2015), systems where microspore embryogenesis is induced by midheat treatment, at 32°C to 33°C (Custers et al., 1994; Testillano et al., 2018b). Nevertheless, no information about endogenous auxin dynamics during microspore embryogenesis is available in monocot species. In the present study, we have analyzed auxin dynamics and its involvement in stress-induced microspore embryogenesis in *H. vulgare*, an *in vitro* process of cell reprogramming induced by cold stress (4°C). Expression patterns of *HvTAR2-like* and *HvPIN1-like* genes were analyzed during barley microspore embryogenesis, as well as changes in IAA localization and accumulation, by using IAA antibodies, which have been proven very useful to detect auxin in various plant cells and species (Forestan et al., 2010; Rodríguez-Sanz et al., 2014; Rodríguez-Sanz et al., 2015; Corredoira et al., 2017; Mettbach et al., 2017). Moreover, to analyze the possible involvement of auxin in the process, the effects of inhibitors of auxin biosynthesis (Kyn), transport (NPA), and action (PCIB) on microspore embryogenesis initiation and progression were also evaluated.

## Materials and Methods

### Isolated Microspore Culture and Embryogenesis Induction

*Hordeum vulgare* L. cv. Igri plants were used as donor plants. Seeds were vernalized in soil for 1 month at 4°C and then transferred 1 month in a plant growth chamber at 18°C for germination and growth; finally, plants were transferred to a greenhouse under 18°C temperature. Spikes containing microspores at the vacuolated stage, the most responsive stage for embryogenesis induction, were collected and surface sterilized by immersion in 5% bleach for 20 min. Embryogenesis induction was performed through isolated microspore culture as previously described (Rodríguez-Serrano et al., 2012). Stress treatment at 4°C for 23 days and microspore *in vitro* culture in liquid KBP medium, which does not contain exogenous auxin in its composition, were performed.

### Expression Analysis by Reverse Transcriptase–Quantitative Polymerase Chain Reaction

Total RNA was extracted from *in vitro* samples using the RNeasy^®^ Plant Micro and RNeasy^®^ Plant Mini kits (Qiagen) according to the manufacturer’s instruction. cDNAs were obtained from 1.5 μg of RNA using the Superscript™ II reverse transcriptase (Invitrogen) according to Solís et al. (2012). Reverse transcriptase quantitative polymerase chain reaction (RT-qPCR) analyses were performed using the SsoAdvanced™ Universal SYBR Green Supermix on the iQ™ 5 Real-Time PCR Detection Sytem (Biorad). For the expression analyses of *HvTAR2-like* gene, the oligonucleotides used were as follows: 5′- CTTTGGTTTTCGATCCGTGT -3′ and 3′- AGACAACAACTCGCAACCAG -5′, from the sequence of the *HvTAR2-like* gene of *H. vulgare* (HORVU3Hr1G016490.5 accession number in https://webblast.ipk-gatersleben.de/). For the expression analyses of HvPIN1-like gene, the oligonucleotides used were as follows: 5′-CCACTTCATCTCCTCCAACG-3′ and 3′- GAGTAGTGCGAGAAGAGGGA -5′, from the sequence of the *HvPIN1-like* gene of *H. vulgare* (HORVU4Hr1G026680.4 accession number in https://webblast.ipk-gatersleben.de/). Conditions of qPCR were as follows: initial denaturation at 95°C for 30 s, followed by 40 cycles of 5 s at 95°C and 30 s at 58°C. After each run, by heating the samples from 65°C to 95°C, a dissociation curve was acquired to check for amplification specificity. Serial dilutions of cDNA were used to determine the efficiency curve of each primer pair according to Solís et al. (2016). As internal reference gene, cyclophilin gene was used. A minimum of three biological and three technical replicates were analyzed. Data were analyzed with the Bio-Rad CFX Manager 3.1 (3.1.1517.0823) (Biorad), using the Livak calculation method. Transcript levels were normalized to vacuolated microspore before stress stage levels. Differences among stages were tested by one-way analysis of variance (ANOVA) followed by Tukey multiple-comparisons test at *P* ≤ 0.05.

### Fixation and Processing for Microscopic Analysis and Immunofluorescence

Samples from culture at different stages (vacuolated microspores, proembryos, and coleoptilar embryos) were collected and fixed overnight at 4°C with 4% paraformaldehyde in phosphate-buffered saline (PBS) pH 7.3, washed in PBS, dehydrated in acetone, and embedded in the acrylic resin Technovit 8100 at 4°C (Bárány et al., 2018). Semithin sections (2 µm thick) were collected on slides. Some sections were stained with toluidine blue and observed under a bright-field microscope, for cellular structure analysis, and others were kept and 4°C until use for immunofluorescence.

Moreover, barley seeds were germinated in wet filter paper for 48 h. Growing root tips of approximately 4-mm length were excised, fixed, and embedded in acrylic resin as previously described for microspore culture samples. Semithin sections of roots were used as positive control of immunofluorescence assays.

### Immunofluorescence and Confocal Analysis

Immunofluorescence was performed on semithin sections of samples from three independent cultures of microspore embryogenesis. A minimum of three immunofluorescence experiments were carried out for each developmental stage analyzed. Semithin sections were blocked by 10% (w/v) fetal calf serum (FCS) in PBS for 10 min and incubated for 1 h with anti-IAA mouse monoclonal antibody (Sigma, cat. no. A0855) diluted 1:100 in 1% bovine serum albumin (BSA) in PBS. After several washing steps in 1% PBS, signal was revealed with Alexa Fluor 488-labeled anti–mouse immunoglobulin G antibody (Molecular Probes) diluted 1:25 in 1% BSA for 45 min in darkness. Finally, sections were counterstained with 1 mg/ml DAPI (4.6-diamino-2-phenylindole) during 10 min, washed in 1% PBS, mounted in Mowiol, and analyzed by confocal laser microscopy (Leica TCS-SP5-AOBS, Vienna, Austria). Maximum projection images were obtained with the software of the confocal microscope (Leica software LCS version 2.5). Confocal microscopy analysis was performed using the same laser excitation and sample emission capture settings for image acquisition in all immunofluorescence preparations, allowing an accurate comparison among signal intensities.

### Controls of Immunofluorescence Experiments

A positive control and two different negative controls were performed. As positive control, anti-IAA immunofluorescence assays were performed on resin sections of growing root tips from germinated barley seeds; growing roots are known to accumulate auxin in their tips. One of the negative controls was performed avoiding the primary anti-IAA antibody in the immunofluorescence assay. The second negative control was carried out by immunodepletion. For this second negative control, the anti-IAA antibody was incubated with a 5 mg/ml IAA solution at 1:2 (v:v) proportion, at 4°C overnight, to block the antibody by its antigen (IAA), and afterward, this preblocked anti-IAA antibody was used as primary antibody for immunofluorescence assays, following the same protocol and conditions described above.

### Quantification of Auxin Immunofluorescence Signal

Quantification of IAA immunofluorescence intensity was performed with ImageJ software over confocal maximum projections, which were obtained as previously described. Structures of different stages (isolated vacuolated microspore, proembryos and coleoptilar embryos) were outlined as ROIs (regions of interest), and we obtained the fluorescence intensity values in arbitrary units. More than 25 structures of each developmental stage from more than three different immunofluorescence experiments and at least three biological replicates were measured. Significant differences among stages were tested by one-way ANOVA followed by Tukey multiple-comparisons test at *P* ≤ 0.05.

### Pharmacological Treatments With Auxin Inhibitors

Three small compounds with known inhibitory effects over endogenous auxin were applied to microspore cultures. The drugs used were kynurenine (Sigma), inhibitor of auxin biosynthesis; NPA (Duchefa), PAT inhibitor; and PCIB (Sigma), auxin action inhibitor. The compounds were added to the microspore culture media by using stock solution of 500 µM in dimethyl sulfoxide (DMSO) for kynurenine, 100 µM in ethanol for NPA, and 100 µM in DMSO for PCIB. Stock solutions of the drugs were added to the media by filtering them with a sterile Ministart filter (Sartorius Biotech) to get the final concentrations 40 and 100 µM kynurenine, 3 and 10 µM NPA, and 3 and 10 µM PCIB. Mock parallel plates of the same cultures were kept as controls.

To assess the drug effects on embryogenesis initiation, the percentage of proembryos after 4 days culture was quantified in treated and control cultures. Quantifications were carried out using micrographs randomly obtained in an inverted microscope from control and treated microspore culture plates , as previously described by us (Berenguer et al., 2017). Proembryos were identified as rounded multicellular structures, still surrounded by the exine, which displayed higher size and density than microspores. Mean percentages of proembryos were calculated from random micrographs obtained from three independent experiments. A minimum of 1,000 proembryos/microspores were counted for treatment.

Significant differences were tested by one-way ANOVA followed by Tukey multiple-comparisons test at *P* ≤ 0.05. Micrographs of plates after 30 days of culture were taken to qualitatively analyze the effects of the treatments on the production of developed embryos.

Additionally, to assess the effect of the inhibitors on auxin accumulation and/or distribution, anti-IAA immunofluorescence assays were performed on samples from microspore cultures treated by the inhibitors.

## Results

### Expression of Auxin Biosynthesis and Transport Genes *HvTAR2-Like* and *HvPIN1-Like* During Microspore Embryogenesis

**Figure 1:**
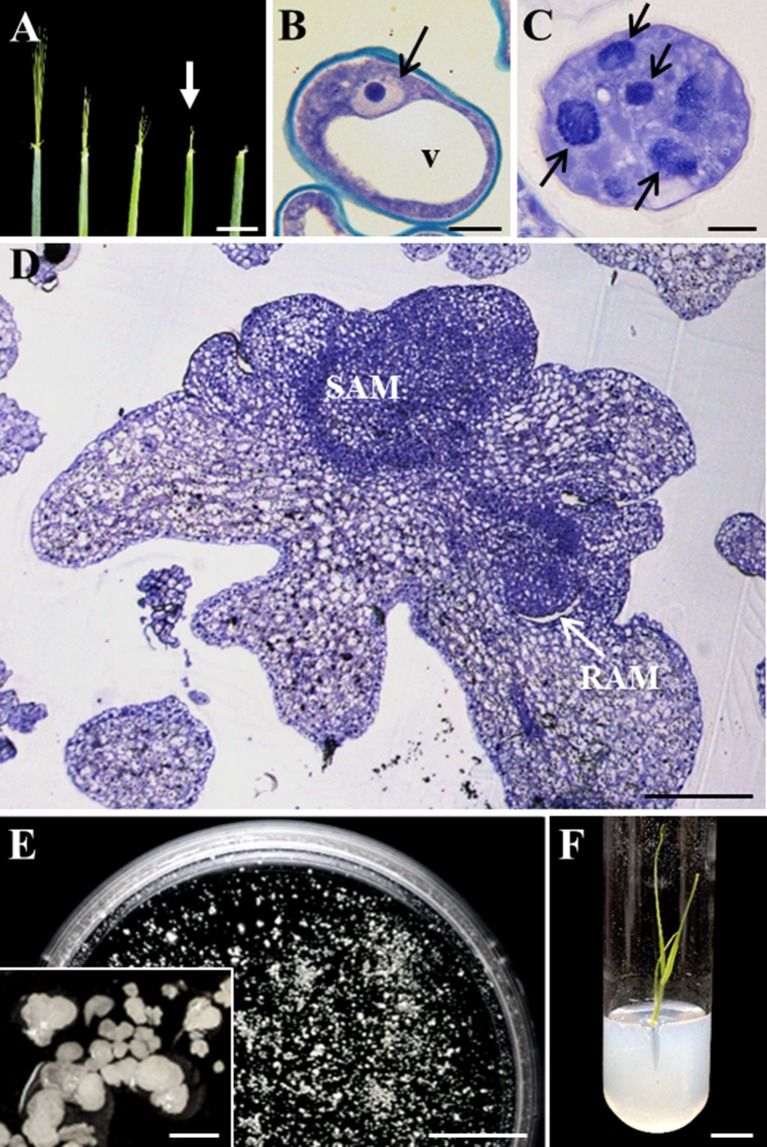
Main stages of stress-induced microspore embryogenesis in *Hordeum vulgare*. **(A)** Immature spikes excised from donor plants at various stages of development. White arrow points to the spike at the most responsive stage, containing vacuolated microspores. **(B**–**D)** Micrographs of semithin sections stained by toluidine blue observed in bright field. **(B)** Vacuolated microspore at culture initiation. **(C)** Proembryo, after 4-day culture. **(D)** Late coleoptilar-leaf stage embryo, after 30-day culture. **(E)** Petri dish containing microspore-derived embryos after 30 days in culture observed under the stereomicroscope, inset: details of several embryos in culture. **(F)** Regenerated plantlet *in vitro*. v, vacuole; black arrows point to nuclei of microspore **(B)** and proembryos **(C)** SAM, shoot apical meristem; RAM, root apical meristem. Bars represent **(A)** 10 mm, **(B**, **C)** 10 µm, **(D)** 200 µm, **(E**, **F)** 1 cm, inset 500 µm.

Microspore embryogenesis was induced in isolated microspore cultures of barley, after cold stress treatment. Immature spikes were collected when the awns appeared outside the upper leaf and showed 10-mm length approximately (**Figure 1A**), which corresponded with the developmental stage of vacuolated microspore (**Figure 1B**), the most responsive stage for embryogenesis induction. After embryogenesis induction by cold stress treatment and a few days in culture, responsive microspores were reprogrammed and divided, forming multicellular structures still surrounded by the exine, the special microspore wall, also named proembryos (**Figure 1C**). Proembryo formation is considered the first sign of embryogenesis initiation. Proembryos further developed and produced globular, transitional, coleoptilar, and leaf stage embryos (**Figure 1D**), which were found after around 30 days in culture (**Figure 1E**). Under appropriate *in vitro* conditions (Rodríguez-Serrano et al., 2012), microspore-derived embryos could germinate and regenerate plantlets (**Figure 1F**).

**Figure 2:**
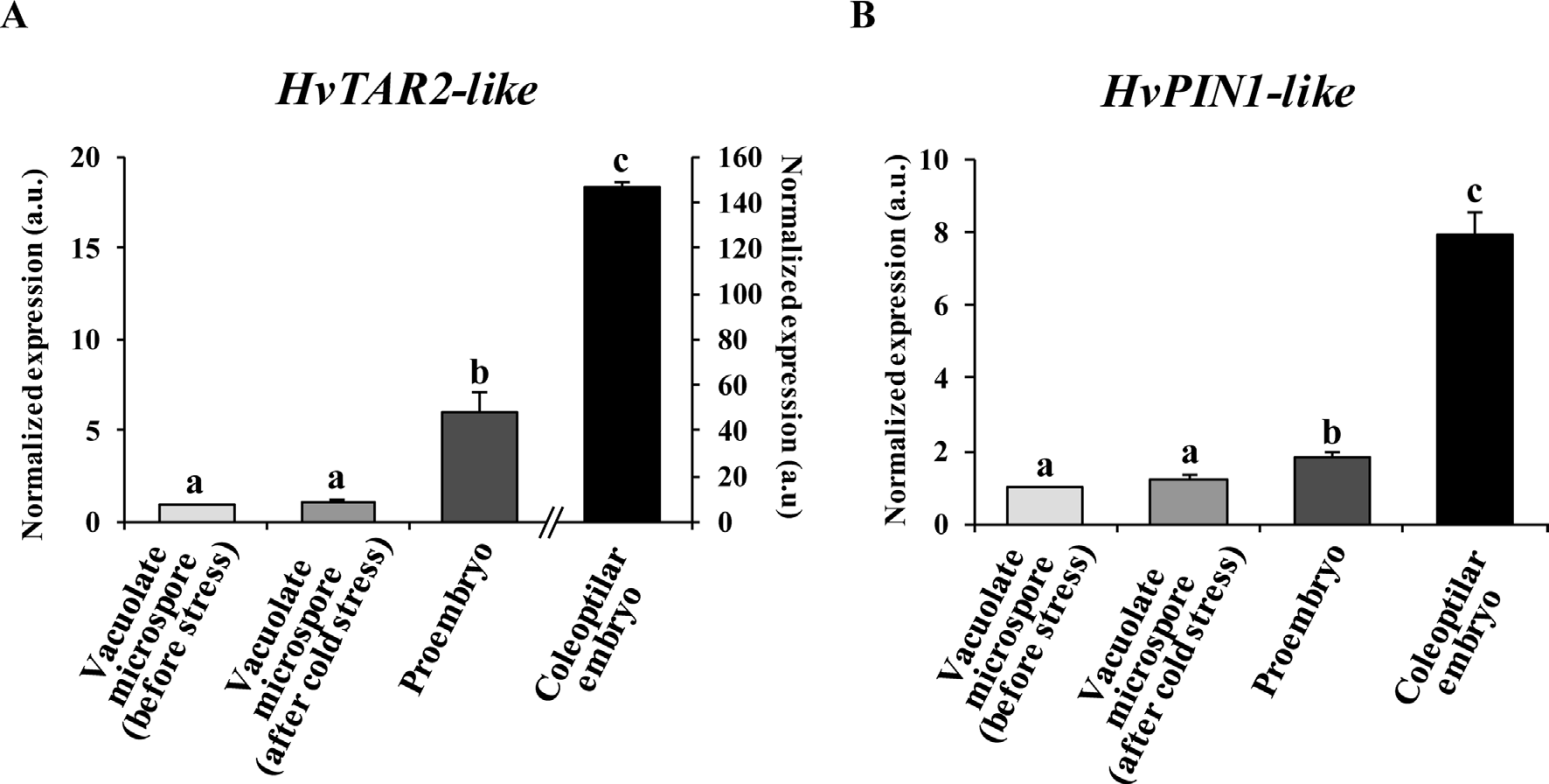
Gene expression patterns of genes *HvTAR2-like* **(A)** and *HvPIN1-like* **(B)** during stress-induced microspore embryogenesis. Histograms showing relative changes of mRNA levels at different developmental stages (vacuolated microspores before and after cold stress, proembryos after 4-day culture and coleoptilar embryos after 30-day culture), normalized to vacuolated microspore levels, as determined by RT-qPCR. Each column represents the mean of at least three biological and three technical replicates. Bars indicate the standard error of the mean (SEM). Different letters indicate significant differences among stages according to ANOVA and Tukey tests at *P* < 0.05.

Expression of auxin-related genes was analyzed at specific stages of the process: a) vacuolated microspores before the stress treatment, b) vacuolated microspores after the stress, c) proembryos, and d) coleoptilar embryos. Because auxin activity depends in many aspects on its local biosynthesis and directional transport among cells, mainly driven by PIN-mediated PAT, in the present study two key genes involved in auxin biosynthesis and polar transport were chosen. First, we selected a tryptophan aminotransferase-related, *HvTAR2-like* gene that functions in the major tryptophan-dependent pathway of auxin biosynthesis. Also, a pinformed1-like, *HvPIN1-like* gene encoding the efflux auxin transporter PIN1, was analyzed. The results showed low levels of *HvTAR2-like* expression in microspores, before and immediately after the inductive stress, and a significant increase of approximately sixfold in the proembryo stage, accompanying embryogenesis initiation (**Figure 2A**). With embryogenesis progression, *HvTAR2-like* expression highly increased. In 30-day-old cultures, coleoptilar embryos exhibited much higher *HvTAR2-like* expression than microspores and proembryos (**Figure 2A**). The expression pattern of *HvPIN1-like* gene was similar to that obtained for *HvTAR2-like*. Microspores before and after the stress treatment showed low expression levels, while *HvPIN1-like* expression significantly increased with embryogenesis initiation, in proembryos, approximately twofold in comparison with microspores (**Figure 2B**). At advanced developmental stages, the expression of the auxin efflux carrier increased much more (near eightfold), in coleoptilar embryos (**Figure 2B**). These results indicated the activation of auxin biosynthesis with microspore embryogenesis initiation and a progressive upregulation with embryo development, in parallel with the activation of PAT.

### Auxin Localization and Accumulation During Microspore Embryogenesis

**Figure 3:**
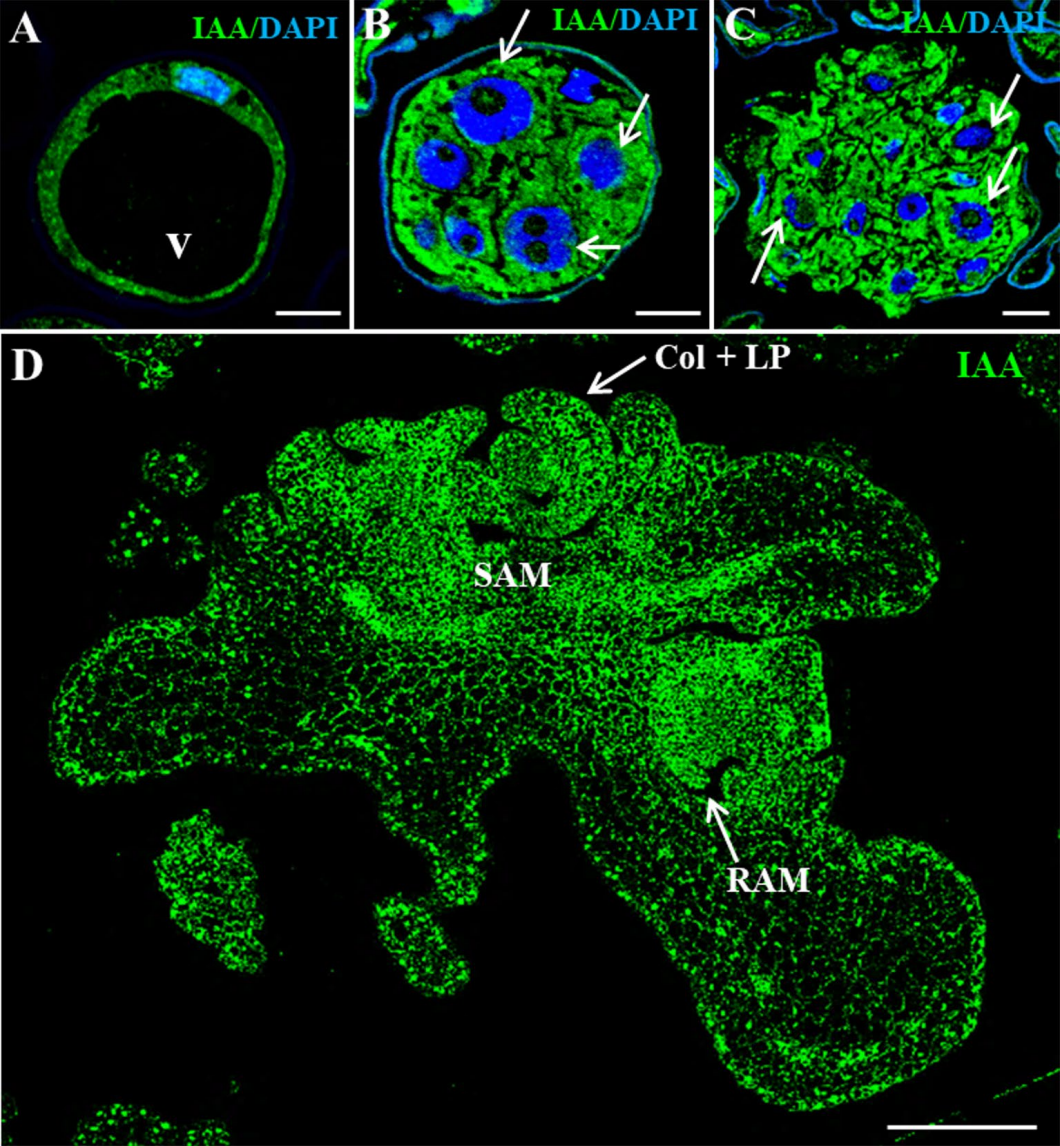
Immunolocalization of IAA during stress-induced microspore embryogenesis. Confocal microscopy images of IAA immunofluorescence (green signal). **(A)** Vacuolated microspore. **(B)** Proembryo, confined by the exine. **(C)** Early globular embryo, just after the exine breakdown. **(D)** Late coleoptilar-leaf stage embryo, after 30-day culture. **(A**–**C)** Merged images of DAPI for nuclei (blue) and IAA immunofluorescence (green). **(D)** IAA immunofluorescence (green). v, vacuole; thin arrows point to nuclei of proembryo cells; SAM, shoot apical meristem; RAM, root apical meristem; col + LP, coleoptile and leaf primordia. Bars represent: **(A**–**C)** 10 µm, **(D)** 200 µm.

Fluorescent protein reporter lines that localize auxin signals or distribution have been widely used to study development and differentiation in the model species *Arabidopsis*, but equivalent tools are still missing for the majority of crop species. Here, to evaluate the changes in auxin distribution and cellular accumulation during barley microspore embryogenesis, immunofluorescence assays followed by confocal imaging were performed, using an anti-IAA antibody. Vacuolated microspores, either before or after the stress, showed low auxin signal intensity in the thin peripheral layer of cytoplasm (**Figure 3A**), being the large central vacuole negative. In contrast, when embryogenesis initiated, proembryos exhibited an intense auxin signal in all their cells (**Figure 3B**), indicating auxin accumulation in proembryo cells. No labeling was found in vacuoles, organelles, and inner cell walls. At later stages, after the exine was broken, early globular embryos also showed high auxin signal that was unevenly distributed among their cells (**Figure 3C**). As embryogenesis progressed, auxin signal increased showing high intensity in coleoptilar and leaf stage embryos, especially in meristems (**Figure 3D**).

**Figure 4:**
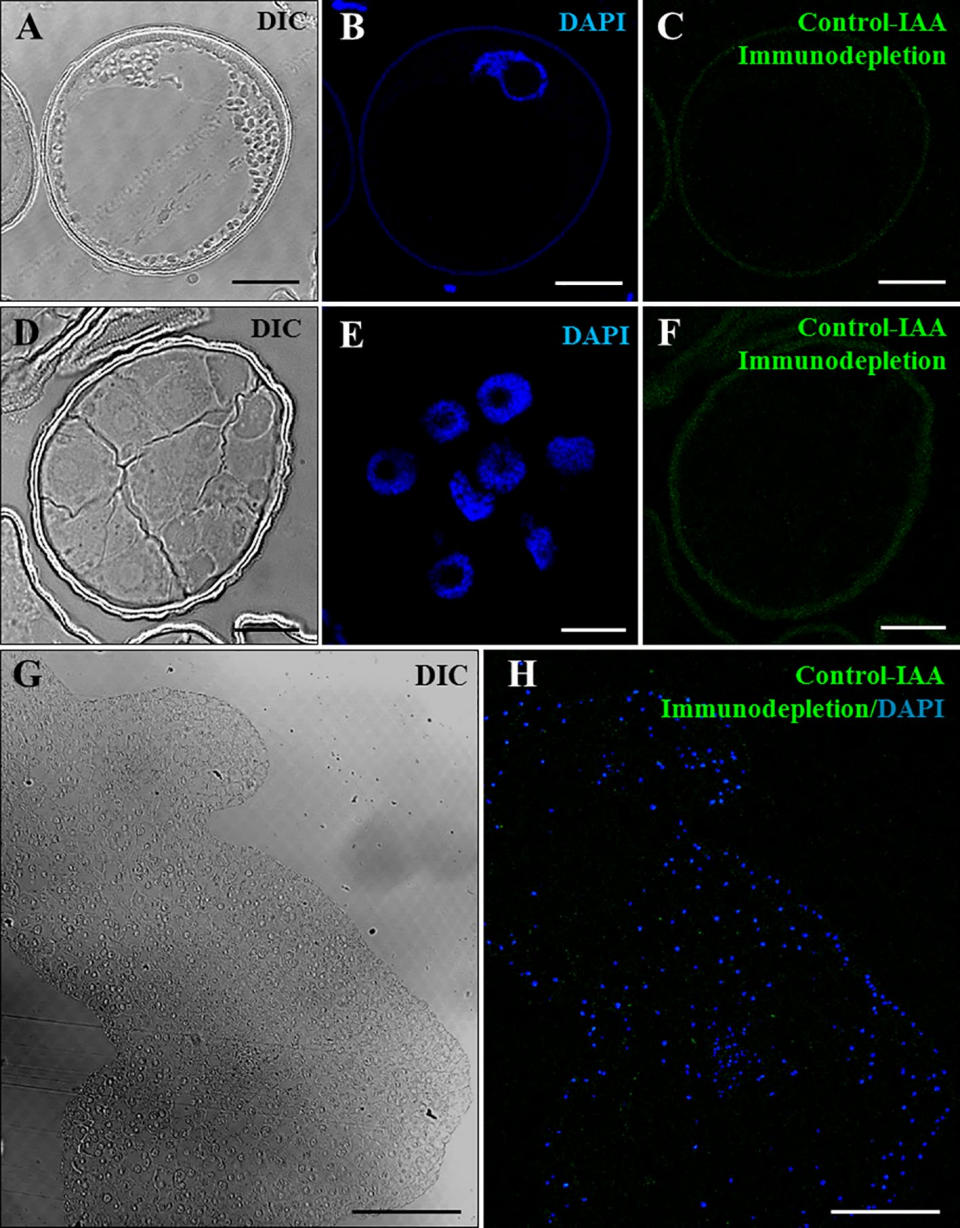
Negative control experiments of IAA immunofluorescence by immunodepletion during stress-induced microspore embryogenesis. Confocal microscopy images of IAA immunofluorescence performed using anti-IAA antibodies pre-blocked with IAA (immunodepletion). **(A**–**C)** Vacuolated microspore. **(D**–**F)** Proembryo, confined by the exine. **(G**, **H)** Region of a late coleoptilar-leaf stage embryo, after 30-day culture. **(A**, **D**, **G)** Differential interference contrast (DIC). **(B**, **E)** DAPI staining of nuclei in blue. **(C**, **F)** IAA immunofluorescence in green. **(H)** Merged image of DAPI for nuclei (blue) and IAA immunofluorescence (green). Bars represent **(A**–**F)** 10 µm, **(G**, **H)** 150 µm.

Several negative and positive control experiments were performed to support the specificity of the antibody and the immunolocalization results. Negative controls performed by eliminating the anti-IAA antibody in the immunofluorescence assays did not provide signal in any sample analyzed (data not shown). Additional negative controls were carried out by immunodepletion assays. First, the anti-IAA antibodies were preincubated with a concentrated IAA solution. During this incubation, the antibodies/immunoglobulins that were specific for IAA reacted with the IAA molecules present in the solution, leading to the blocking of the reactive sites of the anti-IAA antibodies. The preblocked antibody was used for immunofluorescence experiments, which did not shown significant labeling in any developmental stage, vacuolated microspores (**Figures 4A–C**), proembryos (**Figures 4D–F**), or coleoptilar embryos (**Figures 4G, H**). These negative results indicated that the antibodies used were completely preblocked by IAA, and they did not show cross-reactivity with any other antigens present in the samples, supporting the specificity of the antibodies used for IAA localization and the immunofluorescence results.

**Figure 5:**
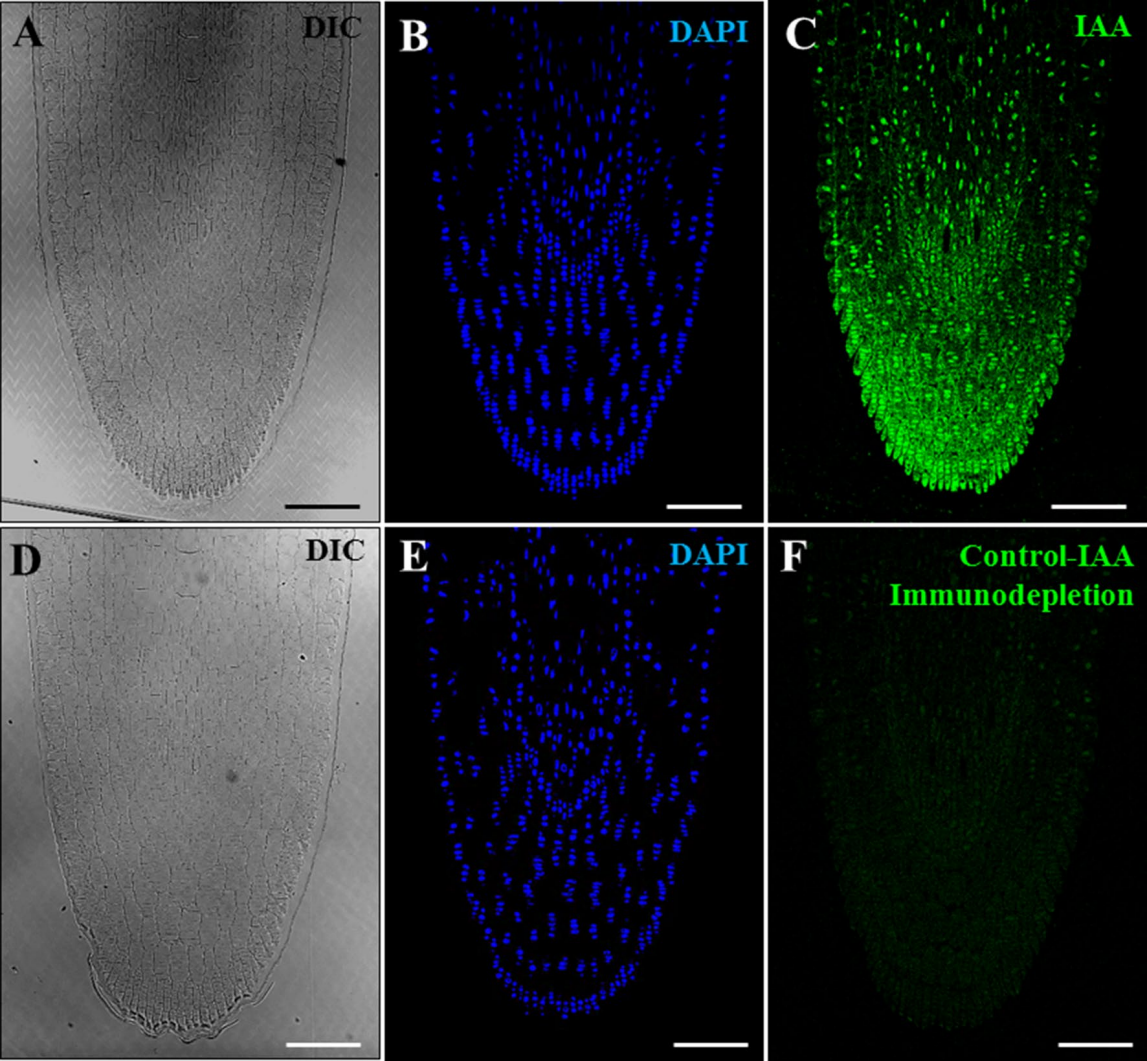
Positive and negative control experiments of IAA immunofluorescence on growing root tips of barley. Confocal microscopy images of IAA immunofluorescence (green signal). **(A**–**C)** Positive control of IAA immunofluorescence on root tips. **(D**–**F)** Negative control of IAA immunofluorescence on roots using anti-IAA antibodies preblocked with IAA (immunodepletion). **(A**, **D)** Differential interference contrast (DIC). **(B**, **E)** DAPI staining of nuclei in blue. **(C**, **F)** IAA immunofluorescence in green. Bars represent 100 µm.

Furthermore, a positive control experiment was performed using growing primary roots, plant organs with reported auxin accumulations localized in their tips. Anti-IAA immunofluorescence assays were carried out on resin sections of growing roots from germinated barley seeds. Results showed an intense immunofluorescence signal on the root tip (**Figures 5A–C**), a region that accumulates auxin. Controls by immunodepletion using preblocked IAA antibodies completely abolished the immunofluorescence signal in the root tips (**Figures 5D–F**). Taken together, the results of the different positive and negative controls performed highly supported the specificity of the antibody and the immunofluorescence results on microspore cultures.

**Figure 6:**
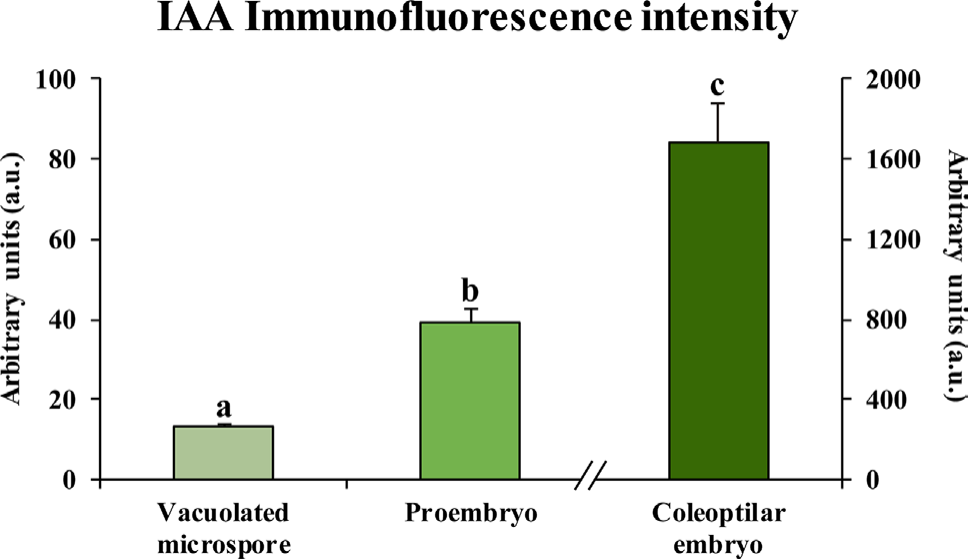
Quantification of IAA immunofluorescence signal intensity during microspore embryogenesis. Histogram represents the level of immunofluorescence intensity, measured in arbitrary units by using ImageJ software tools over confocal maximum projection images at different developmental stages (vacuolated microspores, proembryos after 4-day culture and coleoptilar-leaf stage embryos after 30-day culture). Columns represent the mean of fluorescence intensity (± SEM) of images from at least three immunofluorescence experiments and three biological replicates. Different letters on the columns indicate significant differences according to ANOVA and Tukey test at *P* < 0.05.

Quantification of the auxin fluorescence signal in confocal images captured under the same excitation and capture settings permitted to assess the results of immunofluorescence experiments and provided an estimation of the changes in auxin accumulation at different developmental stages during microspore embryogenesis (**Figure 6**). Before induction, fluorescence intensity of vacuolated microspores was very low, while signal highly increased after induction, in proembryos. At advanced stages, coleoptilar embryos showed a much higher increase in the auxin signal intensity. These results indicated an accumulation of endogenous auxin from the beginning of microspore embryogenesis and its progressive increase during embryo development, a profile that correlated with the expression patterns of genes of auxin biosynthesis, *HvTAR2-like*, and efflux carrier, *HvPIN1-like*.

### Effects of Inhibitors of Auxin Biosynthesis, Transport, and Action on Microspore Embryogenesis

To assess the possible role of auxin in microspore embryogenesis initiation and progression, pharmacological treatments with several auxin inhibitors that interfere with its biosynthesis, polar transport, and action were performed in microspore cultures. Effects of these drugs on the initiation of stress-induced microspore embryogenesis were analyzed by the quantification of the percentage of proembryos in untreated (control) and treated 4-day cultures. The effect of the inhibitors over the progression of embryogenesis was evaluated by comparing the production of coleoptilar embryos after 30 days in treated and control cultures.

**Figure 7:**
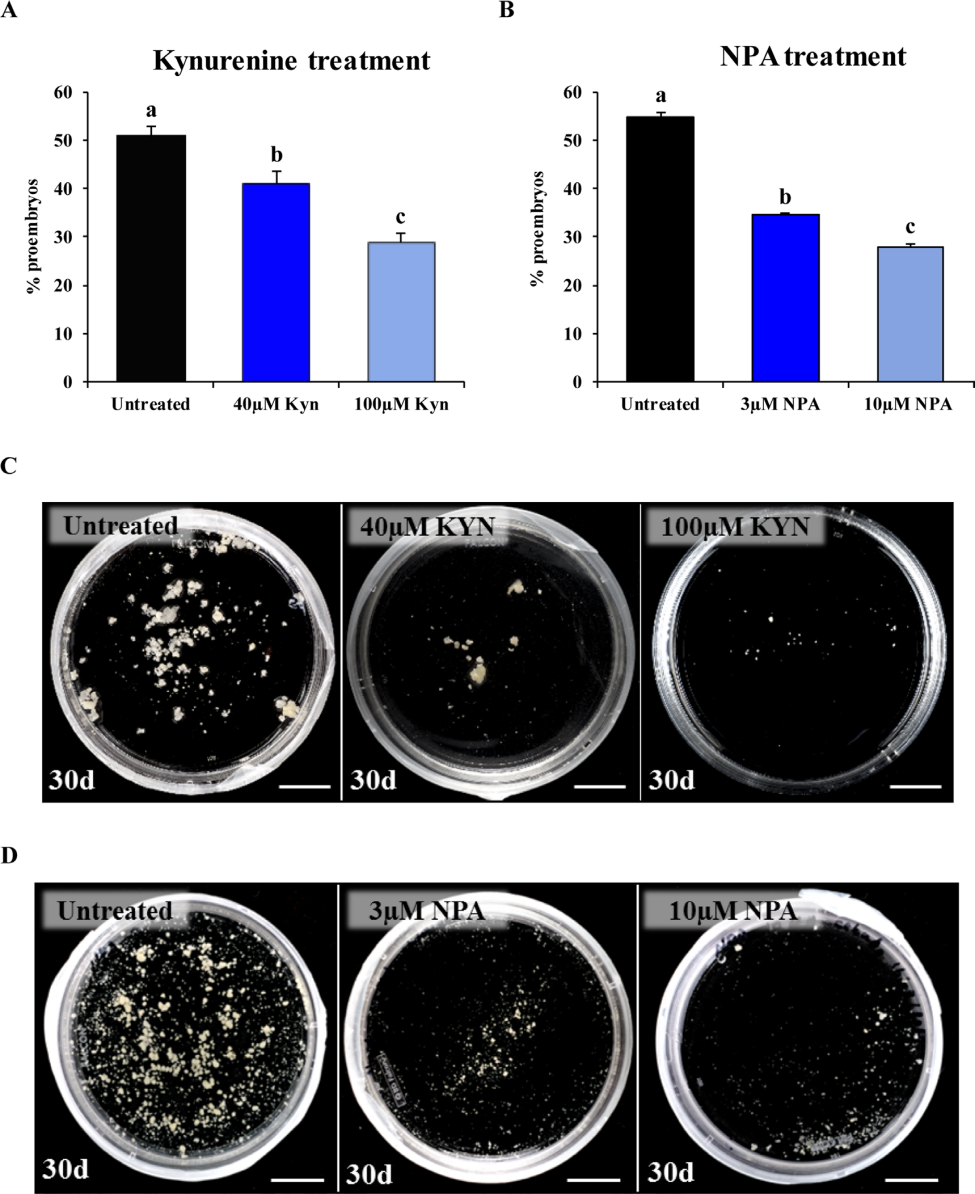
Effect of kynurenine (inhibitor of auxin biosynthesis) and NPA (inhibitor of auxin polar transport) treatments on stress-induced microspore embryogenesis. **(A**, **B)** Quantification of microspore embryogenesis initiation, measured as the percentage of proembryos, in untreated cultures and cultures treated with kynurenine **(A)** and NPA **(B)** at different concentrations (40 and 100 µM kynurenine, 3 and 10 µM NPA). Histograms show the mean percentage of proembryos after 4 days in untreated (control) and treated cultures. Each column represents the mean of three biological replicates. Bars in columns indicate the SEM; different letters on columns indicate significant differences according to ANOVA and Tukey tests at *P* < 0.05. **(C)** Plates showing the microspore-derived embryos produced after 30 days in untreated, 40 µM kynurenine- and 100 µM kynurenine-treated cultures. **(D)** Plates showing the microspore-derived embryos produced after 30 days in untreated, 3 µM NPA-, and 10 µM NPA-treated cultures. Bars represent: 1 cm.

Kynurenine, which inhibits auxin biosynthesis, was applied at 40- and 100-µM concentrations in microspore cultures. In comparison with control cultures, kynurenine-treated cultures showed a reduction in the percentage of proembryos formed after 4 days in culture (**Figure 7A**), in a dose-dependent manner. Mean percentage of proembryos was 50.64% in control cultures, whereas 40 and 100 µM kynurenine treatments reduced it to 39.44% and 28.85%, respectively (**Figure 7A**). After 30 days, control cultures produced numerous embryos; however, cultures treated with kynurenine showed a significant decrease in the number of embryos, being embryo production much lower at 100 µM than at 40 µM (**Figure 7C**).

Microspore cultures were also treated with NPA, an inhibitor of PAT, at 3- and 10-µM concentrations. The mean percentage of proembryos, after 4 days of culture, was significantly reduced in NPA-treated cultures, from 54.76% in control cultures to 34.57% and 28.05% in 3 and 10 µM NPA-treated cultures, respectively (**Figure 7B**). Moreover, the NPA treatments for 30 days drastically reduced the production of differentiated embryos, at the two concentrations applied (**Figure 7D**), indicating that inhibition of PAT severely impaired embryo development.

**Figure 8:**
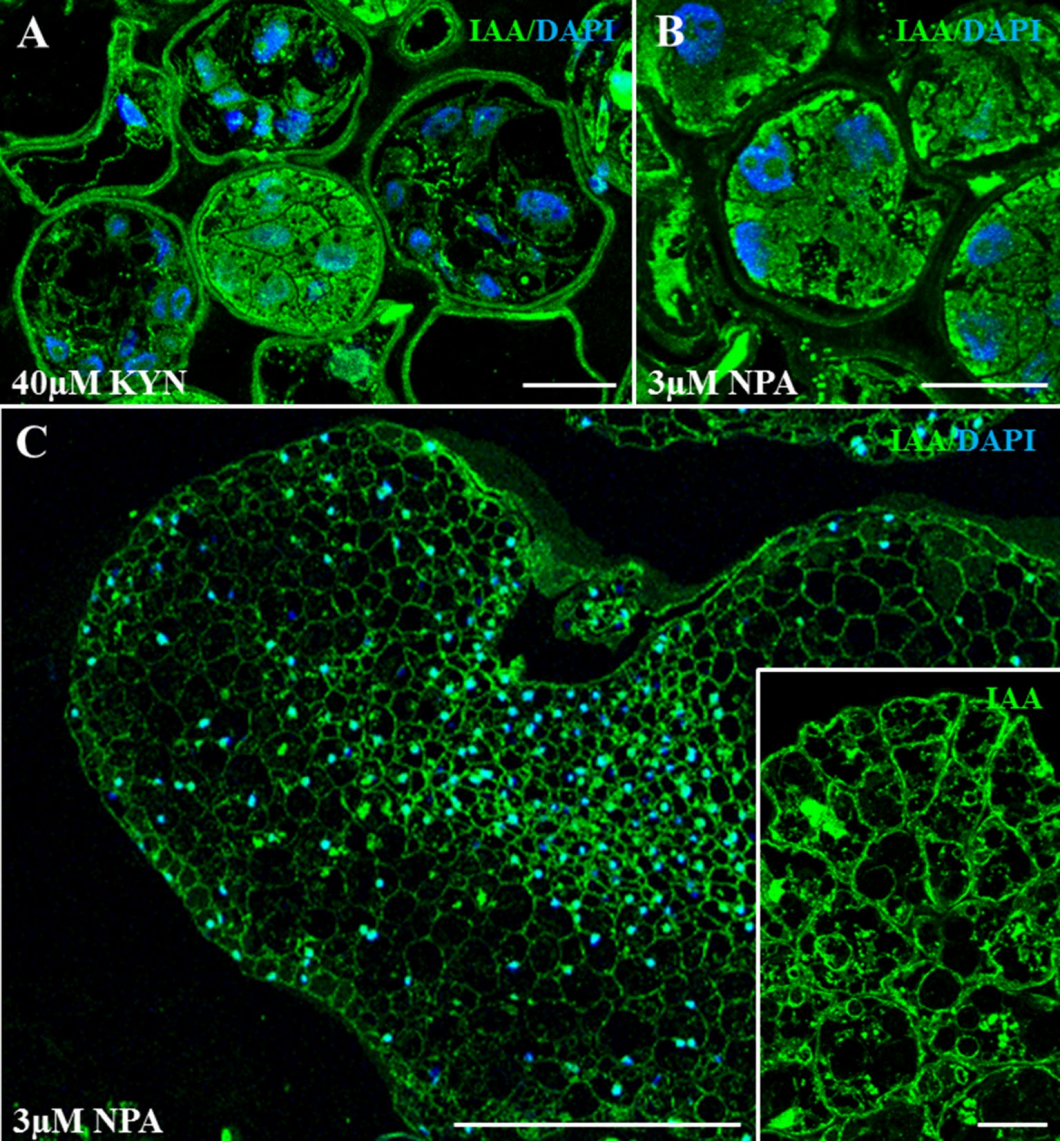
Immunolocalization of IAA in microspore embryogenesis cultures treated with kynurenine (inhibitor of auxin biosynthesis) and NPA (inhibitor of polar auxin transport). Confocal microscopy merged images of IAA immunofluorescence (green signal) and DAPI staining for nuclei (blue signal). **(A)** Proembryos from 40 µM kynurenine-treated cultures, after 4 days in culture. **(B)** Proembryos from 3 µM NPA-treated cultures, after 4 days in culture. **(C)** Developing embryo from 3 µM NPA-treated cultures, after 30 days in culture. Inset: Details at higher magnification of an embryo region from 3 µM NPA-treated cultures. Bars represent **(A**, **B**, **D)** 20 µm, (**C**, inset) 200 µm.

To evaluate the effect of kynurenine and NPA treatments on auxin accumulation or distribution, anti-IAA immunofluorescence assays were performed on samples of treated cultures, at the lowest concentration of inhibitor used. In 40 µM kynurenine-treated cultures, the majority of the proembryos showed low IAA signal on their cells (**Figure 8A**), indicating a decrease in endogenous auxin content, in comparison with control cultures. Kynurenine-treated proembryo cells showed heterogeneous morphologies, with large vacuoles and irregular shapes, very different than proembryo cells from control cultures that exhibited polygonal shape, dense cytoplasms, and intense IAA signal (**Figure 3B**). In kynurenine-treated cultures, only a few proembryos showed the typical cell organization of control cultures and higher IAA signal than the rest (**Figure 8A**). Regarding the treatments with the inhibitor of PAT, in 3 µM NPA-treated cultures, the IAA immunofluorescence signal intensity in proembryos was similar to control cultures; however, some brighter cytoplasmic regions were observed (**Figure 8B**), suggesting heterogeneous accumulations of IAA in the proembryo cells. At later culture stages, the scarce embryos that developed in NPA-treated cultures exhibited irregular morphologies and low IAA signal with an abnormal pattern of accumulation (**Figure 8C**), in comparison with control cultures, suggesting defects in auxin polar transport during embryo development. Moreover, some embryo cells from NPA-treated cultures showed IAA accumulations on small intracellular regions/compartments (inset in **Figure 8C**), suggesting also alterations in intracellular auxin distribution.

**Figure 9:**
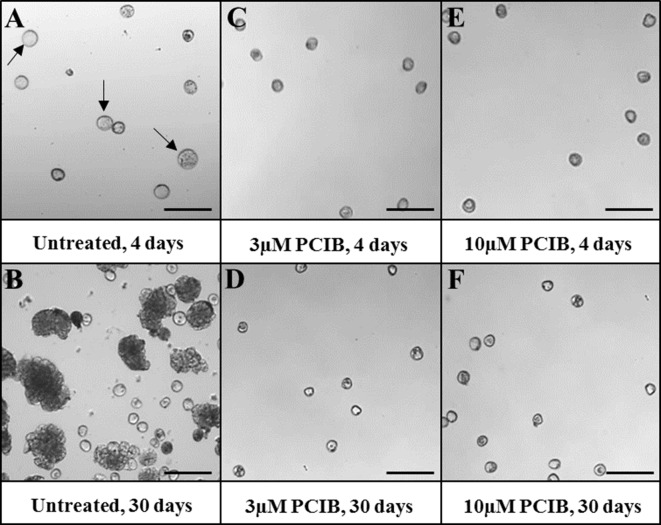
Effect of PCIB (inhibitor of auxin action) treatment in stress-induced microspore embryogenesis. Micrographs showing representative areas of **(A**, **B)** untreated cultures (control), **(C**, **D)** 3 µM PCIB-treated cultures, and **(E**, **F)** 10 µM PCIB-treated cultures. **(A**, **C**, **E)** Seven-day-old cultures. **(B**, **D**, **F)** Thirty-day-old cultures. Arrows point to proembryos (larger, dense-rounded structures) formed after 4 days in untreated cultures. Bars represent 150 µm.

The third drug used was PCIB, known as an auxin antagonist that blocks auxin action and prevents its physiological effects. The application of PCIB treatments to microspore cultures completely blocked embryogenesis from early stages, at 3- and 10-µM PCIB concentrations (**Figure 9**). After 4 days, control cultures produced proembryos, clearly distinguishable from nonresponsive microspores (**Figure 9A**). However, almost no proembryos could be observed in 4-day cultures treated with 3 and 10 µM PCIB (**Figures 9C, E**). After 30 days, numerous embryos were developed in control cultures (**Figure 9B**), while no further development was observed in cultures containing PCIB, at any concentration (**Figures 9D, F**).

## Discussion

From decades, advances in plant embryogenesis *in vitro* protocols have been mostly based on trial-and-error approaches, since the regulating mechanisms underlying the induction of cell reprogramming and conversion into a totipotent embryogenic cell are still elusive. Increasing evidence indicates that plant cell reprogramming, totipotency acquisition, and embryogenesis initiation are regulated by epigenetic and hormonal mechanisms (Smertenko and Bozhkov, 2014; Feher, 2015; Díaz-Sala, 2018; Testillano, 2019). The aim of the present study was to analyze endogenous auxin changes and the role of this phytohormone in stress-induced microspore embryogenesis, model system of plant cell reprogramming, in the monocot barley, where less information is available. A better understanding of the processes involved will help to identify new targets to improve the efficiency of *in vitro* embryogenesis systems, even in recalcitrant species.

### Increase of Endogenous Auxin Is Required for Microspore Embryogenesis Initiation and Progression

Many reports have shown that exogenous hormone treatment, mainly auxin, is critical for triggering somatic embryogenesis and *in vitro* development (Feher, 2015; Nic-Can and Loyola-Vargas, 2016; Díaz-Sala, 2018); however, microspore embryogenesis is mostly induced by stress treatment, without addition of exogenous auxin in the culture media. Interactions between stress signaling and phytohormones, particularly auxin, have been proposed to lead to somatic embryogenesis induction, although the mechanisms behind this are not known (Feher, 2015; Nic-Can and Loyola-Vargas, 2016). The study performed here has revealed changes in auxin cellular content at the initiation and during progression of microspore embryogenesis, modifications that correlated with the expression profile of the key auxin biosynthesis gene *HvTAR2-like*. The results of the IAA localization assays showed an important increase of endogenous auxin associated with the initiation of embryogenesis, in proembryo cells, whereas this phytohormone was very scarce in microspores, before and just after the application of the inductor stress. Correlating with this observation, auxin biosynthesis gene *HvTAR2-like* was induced at embryogenesis initiation; taking into account the key role of TAR2 in the main auxin biosynthesis pathway, our results highly suggest the induction of auxin biosynthesis at the time of embryogenesis initiation and a further increase with embryo development and differentiation.

In the last years, several studies in *Arabidopsis* have revealed that auxin concentration, distribution, and signaling are key factors for cell fate, proliferation, and differentiation and that auxin gradient and asymmetric distribution play an essential role in plant developmental growth and environmental responses, including lateral root initiation; early somatic embryogenesis; meristems initiation (Periañez-Rodríguez et al., 2014; Su et al., 2014); tropic growth, as gravitropism and phototropism (Wang et al., 2013; Zhang et al., 2017); and plant skotomorphogenesis and photomorphogenesis (Yu et al., 2016; Cao et al., 2019). In *Arabidopsis* zygotic embryogenesis, auxin accumulation has been detected in cells of the embryo proper from very early stages (Möller and Weijers, 2009; Robert et al., 2013). More recently, it has been reported that auxin increases in ovules after fertilization, due to increased auxin biosynthesis in the integuments, and this auxin is required for embryo development (Robert et al., 2018). In indirect somatic embryogenesis, plant growth regulators play a central role in both the initiation of proembryogenic masses (PEMs) and the differentiation and maturation of somatic embryos. Endogenous auxin levels have shown to be relatively higher during proliferation of PEMs and somatic embryos of tree species, like Norway spruce, *Abies alba* and *Quercus alba* (Hakman et al., 2009; Corredoira et al., 2017; Vondrakova et al., 2018). These studies indicate that high endogenous auxin levels may be involved in the activation of proliferation of reprogrammed adult cells leading to PEM formation and somatic embryogenesis initiation.

A previous report in *B. napus* microspore embryogenesis, where reprogramming is induced by 32°C treatment, reported *de novo* auxin synthesis with embryogenesis initiation, accompanying the first microspore divisions (Rodríguez-Sanz et al., 2015). Moreover, in *Q. suber*, a system where microspore embryogenesis is induced by midheat stress through anther culture, a differential and significant increase of IAA endogenous levels was found in early multicellular embryo cells, in comparison with microspores (Rodríguez-Sanz et al., 2015). Although, in comparison with *Arabidopsis*, the role of endogenous hormones during early embryogenesis in monocots is still largely unknown, some studies using DR5 auxin reporters in maize have shown that shortly after fertilization auxin activity is detected in the endosperm but not in the early embryo (Chen et al., 2014). Nevertheless, the efficacy of DR5 auxin reporter in barley and other grasses has been recently discussed (Kirschner et al., 2018; Shirley et al., 2019). In the present study, the anti-IAA immunofluorescence assays and confocal imaging, together with the appropriate positive and negative controls performed and the quantification of fluorescence signal intensity, have been convenient and reliable approaches to evaluate changes in endogenous auxin accumulation during the process of microspore embryogenesis. Our results in barley microspore embryogenesis, a system where embryo development is initiated in the absence of surrounding extraembryonic tissue, suggest that the cold stress treatment can trigger the response of endogenous auxin, increasing its biosynthesis and intracellular levels, to determine the new developmental cell fate of the microspores. Moreover, the increase in auxin in early proembryos may be related to the activation of the proliferative activity in the reprogrammed microspore and proembryo cells. These findings in a monocot species also indicate that induction by stress of microspore embryogenesis has the same effects on auxin biosynthesis in several species, eudicots and monocots, independently of the stress applied, cold or heat, suggesting common regulatory hormonal pathways.

Once the embryogenic pathway is triggered, further embryo development also involves additional increase in endogenous auxin levels. Our results have shown that at advanced developmental stages auxin biosynthesis gene *HvTAR2-like* was highly induced, correlating with high signal of auxin detected in microspore-derived coleoptilar embryos. Indole–acetic acid concentration was found to increase during embryo development in wheat (Fischer-Iglesias et al., 2001) and maize (Forestan et al., 2010). In *B. napus* microspore embryogenesis, differentiation of embryo is accompanied by increasing levels of auxin and upregulation of *TAA1* biosynthesis gene (Rodríguez-Sanz et al., 2015).

Although the results did not provide a direct evidence of the role of *HvTAR2-like* gene in auxin accumulation during microspore embryogenesis, the similarity between the temporal patterns of both *TAR2-like* expression and endogenous auxin levels highly suggested it. Moreover, in the present study, functional analyses were performed by a chemical biology approach to inhibit biosynthesis and activity of auxin. Kynurenine is an alternative substrate of TAA1/TAR enzymes that selectively inhibits their enzymatic activities (He et al., 2011). When kynurenine treatment was used in embryogenic microspore cultures, the IAA immunofluorescence signal decreased in samples from treated cultures, suggesting that kynurenine was inhibiting auxin biosynthesis in barley microspore cultures, as reported in other plant species and systems (He et al., 2011; de Wit et al., 2015; Nomura et al., 2015). The kynurenine treatment affected the process of microspore embryogenesis at two time points. At the beginning of the process, kynurenine reduced proembryo formation, indicating an important role for auxin in embryogenesis initiation. Later in development, during embryo differentiation, inhibition of auxin biosynthesis by kynurenine led to a significant reduction of fully developed embryo production, meaning that *de novo* auxin biosynthesis is required for embryo development and differentiation during microspore embryogenesis in barley. Moreover, treatments with the auxin antagonist PCIB (Xie et al., 2000; Ma et al., 2018) produced a severe impairment of microspore embryogenesis, from early stages. This indicates that not only auxin biosynthesis, but also its perception and signaling, is necessary for microspore embryogenesis initiation and progression.

### Polar Auxin Transport Is Necessary for Proper Microspore-Derived Embryo Development

Auxin function and availability by cells depend not only on its local biosynthesis, degradation, and conjugation, but also on its polar transport from cell to cell, being auxin efflux carriers of the PIN family crucial players. The analyses performed here on the expression pattern of *HvPIN1-like* gene during barley microspore embryogenesis showed an increase of gene transcription with embryogenesis initiation, in proembryos, and a further increase at advanced developmental stages, in coleoptilar embryos. In *Arabidopsis* zygotic embryogenesis, PIN1 has a central role in the control of the PAT in the developing embryo. At early embryogenesis stages (proembryo and early globular stage), auxin is transported from the suspensor cells by PIN7 to the embryo where PIN1 is responsible for the homogeneous distribution of auxin among embryo cells (Petrasek and Friml, 2009). Later in development, PIN1, together with PIN7 and PIN4, plays a central role in polarizing auxin fluxes to establish auxin gradients in specific embryo regions (Petrasek and Friml, 2009; Zazimalova et al., 2010). During microspore embryogenesis of the eudicot *B. napus*, auxin efflux carrier PIN1-like gene expression is induced at early stages and increases during embryo development (Rodríguez-Sanz et al., 2015). PIN auxin efflux carriers have been also identified in monocots, with central roles in regulating polar transport and auxin accumulating patterns during development, as well as during embryogenesis (Forestan and Varotto, 2012; Balzan et al., 2014; Locascio et al., 2014). In maize, ZmPIN1-mediated auxin transport controls differentiation and patterning during zygotic embryogenesis (Forestan et al., 2010). Our results have shown an expression pattern of *HvPIN1-like* similar to that reported in maize zygotic embryogenesis, in which *ZmPIN1* is involved in the heterogeneous auxin distribution at advanced developmental stages leading to auxin accumulation in specific regions, like shoot and root apical meristems (Forestan et al., 2010). Although no direct evidence is provided in the present study on the involvement of *HvPIN1-like* in auxin accumulation and distribution patterns during microspore embryogenesis, given the induction of its expression during embryo development and its reported roles in zygotic embryogenesis of other grasses, it could be hypothesized that PIN1-like gene would participate in the auxin distribution during microspore embryogenesis. Our results also revealed that at advanced developmental stages auxin accumulated in some regions of developing embryos, likely meristems, providing evidence of the relevance of PAT in microspore embryogenesis of barley, as occurs in zygotic embryogenesis.

Additional evidence of the involvement of PAT in the correct progression of microspore embryogenesis was obtained by pharmacological assays with the auxin transport inhibitor NPA. This small compound has been used to inhibit PIN-mediated PAT in many different plant developmental processes (Nishimura et al., 2011; Forestan and Varotto, 2012; Balzan et al., 2014; Teale and Palme, 2018). During zygotic embryogenesis, NPA application leads to embryo patterning defects in several plant species, supporting the requirement of PAT for normal embryo development in eudicots (Weijers and Jurgens, 2005), as well as in monocots (Fischer-Iglesias et al., 2001; Forestan et al., 2010; McSteen, 2010; Shirley et al., 2019). N-1-naphthylphthalamic acid has been reported to have various targets besides PIN1, with inhibitory effects on auxin transport (Nagashima et al., 2008; Peer et al., 2009). In *Arabidopsis*, it has been shown that the NPA treatment impairs PIN endocytosis and localization and thereby affects PIN-mediated asymmetric auxin distribution during hypocotyl phototropism and hook formation (Yu et al., 2016; Zhang et al., 2017). Although the identification of NPA action mode in microspore embryogenesis requires further investigation, we could hypothesize that probably NPA may affect microspore embryogenesis initiation through impairment of PIN polar localization and/or intracellular trafficking and thereby auxin distribution. Our results in microspore cultures treated with NPA clearly showed reduction of proembryos and differentiated embryos, as well as abnormal auxin distribution in embryo cells; these findings allow confirming the need of active auxin transport during microspore embryogenesis, supporting the involvement of auxin transport in correct *in vitro* embryo initiation and development.

## Conclusions

The analyses performed here have revealed not only that induction of auxin biosynthesis gene and intracellular auxin accumulations are associated with the initiation of stress-induced microspore embryogenesis, but also the requirement of auxin biosynthesis for correct *in vitro* embryo development, from initial until advanced stages. The results also demonstrate that concomitantly with auxin synthesis, PAT, likely through efflux carrier *HvPIN1-like*, is induced with microspore embryogenesis, being essential for *in vitro* embryo formation. These findings indicate that induction by stress of microspore embryogenesis has the same effects on auxin biosynthesis in a monocot species than in eudicots, independently of the stress applied, cold or heat, what suggests a common and central role of auxin in the regulation of the process. They also provide new evidence of the involvement of PAT in *in vitro* embryo initiation and development, as occurs in zygotic embryogenesis, in a monocot plant, where less information is available. This provides new insights into the regulating mechanisms of microspore embryogenesis in cereals, opening up new possibilities to improve its efficiency for DH production in breeding programs.

## Data Availability Statement

The datasets generated for this study are available on request to the corresponding author.

## Author Contributions

YP-P performed most of the experimental work. YP-P carried out microspore cultures, microscopy analyses, immunofluorescence assays and quantification of signal, pharmacological *in vitro* treatments with kynurenine, and gene expression analyses; prepared the figures; and contributed to the writing of the figure legends and *Materials and Methods* sections. A-AE-T performed microspore cultures and *in vitro* treatments with NPA and PCIB and contributed to some immunofluorescence assays. M-TS performed the search and selection of gene sequences, designed the primers, and contributed to some RT-qPCR assays. MR participated in the discussion of the results. PST conceived, designed, and supervised the experimental work; analyzed the results; elaborated the conclusions; and wrote the manuscript. All authors read and approved the final manuscript.

## Conflict of Interest

The authors declare that the research was conducted in the absence of any commercial or financial relationships that could be construed as a potential conflict of interest.
